# Fruit Flies (Diptera: Tephritidae) in Minas Gerais, Brazil: Trophic Interactions and New Reports

**DOI:** 10.3390/insects16010017

**Published:** 2024-12-28

**Authors:** Rosamara Souza Coelho, Clarice Alvarenga, Marvin Pec, Ana Luisa Rodrigues-Silva, Pedro Maranha Peche, Emanoel Alves, Rosangela Marucci

**Affiliations:** 1 Department of Entomology, Federal University of Lavras (UFLA), Lavras 37200-900, MG, Brazil; rosamaracoelho@gmail.com (R.S.C.); aninhamj97@gmail.com (A.L.R.-S.); emanoelcost@hotmail.com (E.A.); 2 Department of Agricultural Sciences, State University of Montes Claros (UNIMONTES), Janauba 39448-581, MG, Brazil; 3 Department of Entomology and Acarology, Luiz de Queiroz College of Agriculture (ESALQ/USP), University of São Paulo, Piracicaba 13418-900, SP, Brazil; pecmarvin@gmail.com; 4 Department of Agriculture, Federal University of Lavras (UFLA), Lavras 37200-900, MG, Brazil; pedro.peche@ufla.br

**Keywords:** host fruits, *Ceratitis capitata*, *Anastrepha*, parasitoids

## Abstract

A diverse orchard with fruit fly hosts may provide information about trophic relationships, including new insights into beneficial insects. We evaluated the composition of the fruit fly complex to provide information on tephritid species, parasitoids and multitrophic interactions for the southern region of Minas Gerais, Brazil. Sampling was carried out using traps and by collecting fruits from plants and/or the ground according to availability/the fruiting period. Occurrences of *Anastrepha amita* Zucchi and *A. punctata* Hendel were recorded for the first time in the state of Minas Gerais, and new trophic associations were obtained for *A. bahiensis* Lima, *A. bistrigata* Bezzi, *A. fraterculus* (Wiedemann), *A. obliqua* (Macquart) and *Ceratitis capitata* (Wiedemann). Ten tephritid species were obtained from trap sampling, with *C. capitata*, *A. fraterculus* and *A. obliqua* being the most abundant. Five species of fruit flies and seven species of parasitoids were obtained from fruits. The braconid *Doryctobracon areolatus* (Szépligeti) was the most frequently collected among the parasitoid species. Pitanga (*Eugenia uniflora* L.) and purple guava (*Psidium myrtoides* O. Berg) fruits were classified as repositories of fruit fly parasitoids.

## Simple Summary

Fruit flies are pests of major global importance, infesting a variety of fruit species of economic importance. Assessing host plant interactions, fruit flies species composition and natural enemies is essential to promote the successful management of these pests. From this perspective, the objective of this study was to determine the fruit fly species, their natural enemies (parasitoids) and their interactions with host plants. Sampling was carried out with traps and by collecting fruits directly from plants and from the ground, according to availability. Ten species of fruit flies were collected with traps, and five species of fruit flies and seven species of parasitoids were obtained from the collected fruits. The parasitoid *Doryctobracon areolatus* was the most frequently collected. Pitanga and purple guavas were classified as repositories of fruit fly parasitoids.

## 1. Introduction

Plant characteristics influence herbivores, directly and indirectly, affecting natural enemies [1]. For example, the behavior of herbivores can be modulated by chemical signals emitted by host plants [2,3,4,5], as well as morphological configurations (including fruit color, firmness and pericarp thickness) [6,7], nutritional rewards [8] and innate and/or acquired attraction [9,10]. Likewise, plants can alter the behaviors of natural enemies to reduce or increase herbivory [1,11,12,13,14]. In particular, the interactions between frugivorous tephritids (Diptera: Tephritidae) and parasitoids have received considerable attention, mainly due to the economic relevance and advancement of biological control [15,16,17]. In this regard, understanding the multitrophic interactions of fruit flies may provide new insights and approaches for integrated management, especially the biological control of tephritids.

*Anastrepha* spp. and *Ceratitis capitata* (Wiedemann) (Diptera: Tephritidae) infest a variety of fruit species, regardless of their economic importance. *Anastrepha* spp. are native to the American continent, represented by 128 species in Brazil and associated with 322 host plant species in 60 families [18]. In contrast, *C. capitata* is native to sub-Saharan Africa [19] and is the only species of the genus present in Brazil, associated with 117 host species in 31 families [20]. In the field, these flies are often parasitized by hymenopterans of the families Braconidae and Figitidae [21,22]. Koinobiont species parasitize larvae and emerge from the pupae of their hosts. Among the native parasitoids, *Doryctobracon areolatus* (Szépligeti) (Hymenoptera: Braconidae) stands out and is often associated with *Anastrepha* spp. [23].

The state of Minas Gerais is included in the domain of three Brazilian biomes: Cerrado, Atlantic Forest and Caatinga. Despite the importance and diversification of fruit crops, studies involving fruit fly surveys have mostly focused on the northern region [24,25,26,27] and the Zona da Mata Mineira region [28]. Interestingly, little sampling effort has been devoted to the southern region [29], where information on the multitrophic interactions of tephritids remains scarce. Considering the high polyphagia and phenotypic plasticity of fruit flies, coupled with the lack of knowledge of the species occurring in the southern region of the state, sampling via fruit collection is essential to understand these relationships.

In this study, we test the hypothesis that fruit species influence fruit fly and parasitoid communities in a diverse orchard. Furthermore, infestation and parasitism respond to variations in host fruits. From this perspective, our objective was to determine the composition of the fruit fly complex to provide information on the species of tephritids and parasitoids and their multitrophic interactions in the southern region of Minas Gerais. The interactions were evaluated for the following host tree species: *Eugenia stipitata* MacVaugh, *Psidium myrtoides* O. Berg, *Campomanesia xanthocarpa* O. Berg, *Psidium guajava* (L.), *Plinia jaboticaba* (Vell.) Kausel, *Eugenia involucrata* Dc, *Syzygium jambos* (L.) Alston, *Eugenia uniflora* L., *Eugenia pyriformis* Cambess, *Coffea arabica* L., *Averrhoa carambola* L., *Cirtius paradisi* Macfad, *Citrus reticulata* Blanco, *Citrus aurantium* L., *Citrus sinensis* (L.) *Fortunella margarita* Swingle, *Citrus limonia* Osbeck, *Poncirus trifoliata* (L.), *Citrus unshiu* Marcov., *Eriobotrya japonica* (Thunb.), *Pyrus communis* L. and *Prunus persica* (L.). Understanding these interactions will facilitate the implementation of assertive fruit fly control strategies in orchards.

## 2. Materials and Methods

### 2.1. Study Area

The study was conducted in the experimental orchard of the Federal University of Lavras (UFLA), Minas Gerais, Brazil (44°58′56″ W and 21°13′50″ S, altitude 908 m). According to the Köppen classification, the climate of the region is a Cwa subtropical climate, that is, an altitude tropical climate characterized by a dry winter and a hot, humid summer [30]. The mean annual temperature during the two sampling years (February 2019–June 2021) ranged from 15 to 23.4 °C (minimum temperatures from 10.3 to 19.9 °C and maximum temperatures from 16.3 to 30.4 °C), with total annual rainfall amounts of 1124 mm (2019), 1633.9 mm (2020) and 661.5 mm (January to June 2021) and average relative humidity ranging from 50.74 to 78.65% (Figure S1). The meteorological data were obtained from a meteorological station located on the UFLA campus. The area covers 17 ha of diverse vegetation, with several types of cultivated fruit trees that host fruit flies throughout the year but at different times, depending on the fruiting period. The university orchard consists of collections and experimental areas. The collections include pear trees (6 cultivars); medlar trees (1 cultivar); fig trees (18 cultivars); quince trees (27 cultivars); small fruits such as black mulberry, raspberry and physalis (1 cultivar each); grapevines (17 cultivars); dragon fruit (6 cultivars); and citrus trees (10 cultivars). The orchard also has a collection of native and exotic plants of lesser economic importance, with a total of 40 species with one or two individuals each (Table S1). There are also forest fragments, in addition to urban areas around the campus [31]. Fruits infested with fruit flies were also sporadically collected in domestic orchards in the rural areas of the municipalities of Ijaci, Minas Gerais, Brazil (44°55′43″ W and 21°10′15″ S, altitude 833 m), and Itumirim, Minas Gerais, Brazil (44°52′15″ W and 21°19′01″ S, altitude 870.56 m), to compose the species list and establish trophic relationships.

### 2.2. Sampling

#### 2.2.1. Fruit Collection

Fruit sampling occurred from February 2019 to June 2021 and varied over time, depending on the availability/fruiting season. Mature fruits or fruits in the process of ripening were collected randomly from the trees and/or the ground if they had fallen, and they were placed in labeled plastic trays (435 × 285 × 80 mm) and sent to the laboratory. The fruits of each species (separated into tree and ground samples) were sequentially weighed, counted and kept in plastic trays with a thin layer of vermiculite as a substrate for pupation. These containers were covered with voile fabric and kept under controlled conditions (25 ± 2 °C, 50 ± 10% RH and 12 h photophase). After a period of 15–20 days, the vermiculite was examined, and the pupae were counted and placed in new labeled plastic containers (50 mL) containing vermiculite and maintained in this condition until the emergence of adult flies and/or parasitoids. Finally, the obtained adults were counted, sexed and preserved in 70% alcohol for later identification.

#### 2.2.2. Traps

Three McPhail traps containing bait composed of hydrolyzed corn protein (Bio Anastrepha^®^) diluted in water to 5% were used. The traps were installed in the tree canopy approximately 1.50 m above the ground and inspected fortnightly, at which time the captured fruit fly specimens were collected, and the food attractants were replaced. The specimens were washed with water in a sieve and carefully placed in plastic containers (250 mL) with 70% alcohol, labeled and sent to the laboratory for screening. The traps were maintained uninterruptedly from November 2019 to April 2021 (Figure S2).

### 2.3. Identification of Insects

The identification of fruit flies and parasitoids was based exclusively on adults. The specific identification of *Anastrepha* was based only on females [32,33], and that of *C. capitata* was based on both males and females. The species of Braconidae were identified according to [34,35], and those of Figitidae were identified according to [36]. *Anastrepha fraterculus* (Wiedemann) corresponds to a complex of cryptic species; however, it was considered lato sensu in this study. Reference samples of fruit flies and parasitoids were preserved in 70% alcohol and later deposited at the Museum of Entomology of Luiz de Queiroz College of Agriculture (ESALQ, USP; voucher codes ESALQENT001810 to ESALQENT001839).

### 2.4. Data Analysis

All analyses were performed using R 4.0 software [37]. We performed a descriptive analysis of the host and nonhost fruit species of fruit flies, as well as the species of flies and parasitoids obtained from these samples. The tephritid infestation index was calculated for samples of each host plant species by dividing the total number of pupae obtained from the fruit sample by the number of fruits. The rate of parasitism was determined by dividing the total number of emerged parasitoids by the sum of parasitoids and flies that emerged from the sample and multiplying by 100 [24]. For the analysis, we only used the specimens obtained from fruits collected in the experimental orchard since there was a periodicity in the sampling of these fruits. The total species richness was calculated from the number of individuals collected (for flies and parasitoids) by grouping all samples together to obtain the Coleman rarefaction curve and the bootstrap richness estimator. For the species composition analysis of the flies and parasitoids obtained from fruits sampled from trees and/or the ground, a permutational multivariate analysis of variance (PERMANOVA) was performed using the “Vegan” package [38]. The interactions of fruit flies with host fruits and of parasitoids with infested fruits were presented in a heatmap to determine the relationships between species (fruit flies and/or parasitoids) and fruit species. We showed which fruits contributed to the abundance and diversity of species in the study area. The strength of species interactions between fruits was measured by the Euclidean distance.

The frequency was calculated from the proportion of individuals of a species in relation to the total number sampled. The total richness of the species collected in traps was calculated from the number of individuals by grouping the three traps for 18 months of sampling to generate the Coleman rarefaction curve and the bootstrap richness estimator.

### 2.5. Population Fluctuation

To visualize the different distribution patterns of the fruit fly species, the population fluctuation was established using only the female fruit flies captured monthly in the traps. In this analysis, the three most abundant fruit fly species were considered.

## 3. Results

### 3.1. Fruit Collection

#### 3.1.1. Fruit Flies

Fruits from 30 species were sampled, distributed in nine botanical families, totaling 166.71 kg and 8146 fruits collected. Among the obtained fruit samples, we recorded the presence of *Anastrepha* spp. and/or *C. capitata* in 23 species belonging to the Myrtaceae, Rubiaceae, Oxalidaceae, Rutaceae and Rosaceae families. The index of fruit fly infestation varied among fruits obtained from trees and soil and among fruit species (Table S1). In addition, the highest average infestation rates were obtained in *Eugenia stipitata* MacVaugh (7.67), *Prunus persica* (L.) cv. Libra (6.73) and *Psidium guajava* (L.) cv. Purple (6.27) and cv. Paluma (5.71) (Table S1).

Of all the species, the South American fruit fly, *A. fraterculus*, infested the largest number of hosts, including 20 fruit species distributed among the five botanical families sampled. In particular, the plant species *Campomanesia xanthocarpa* O. Berg., *Plinia jaboticaba* (Vell.) Kausel, *Citrus paradisi* Macfad × *Poncirus trifoliata* L. Raf., *Citrus aurantium* L., *Citrus sinensis* (L.), *Citrus limonia* Osbeck, *Poncirus trifoliata* (L.) and *Citrus unshiu* Marcov. were exclusively infested by *A. fraterculus*. The fruit fly *Anastrepha obliqua* (Macquart) was associated with 10 plant species of the five botanical families and was the only species infesting *Eugenia pyriformis* Cambess. *Anastrepha bistrigata* Bezzi was obtained from two species of Myrtaceae. On the other hand, *Anastrepha bahiensis* Lima was obtained only from *Eriobotrya japonica* (Thunb.). Finally, the Mediterranean fly, *C. capitata*, was found in 11 species of the five families sampled (Table 1).

**Figure 1:**
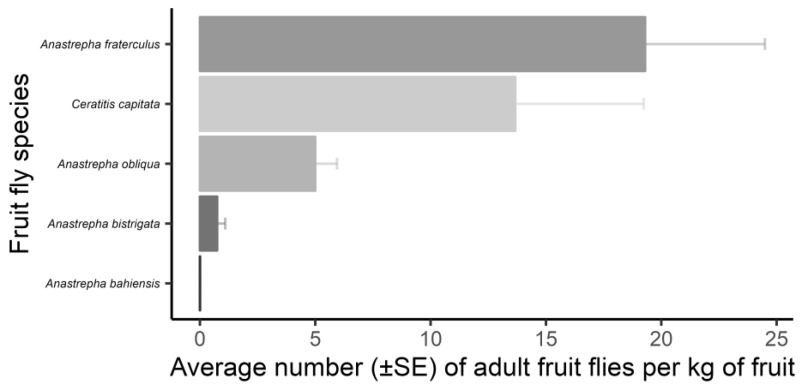
Fruit fly females (Diptera: Tephritidae) per total fruit mass collected in an experimental orchard of the Federal University of Lavras (UFLA), Minas Gerais, Brazil, during the period February 2019 to June 2021. Bars represent the mean number of captured specimens, and lines represent the standard error.

The composition of fruit flies obtained from the fruits collected from the trees and the ground did not differ, suggesting similarity according to the PERMANOVA analysis (*F*_1,38_ = 1.736; *p* < 0.122). The species *A. fraterculus* and *C. capitata* exhibited the highest number of specimens/kg of fruit (Figure 1).

**Figure 2:**
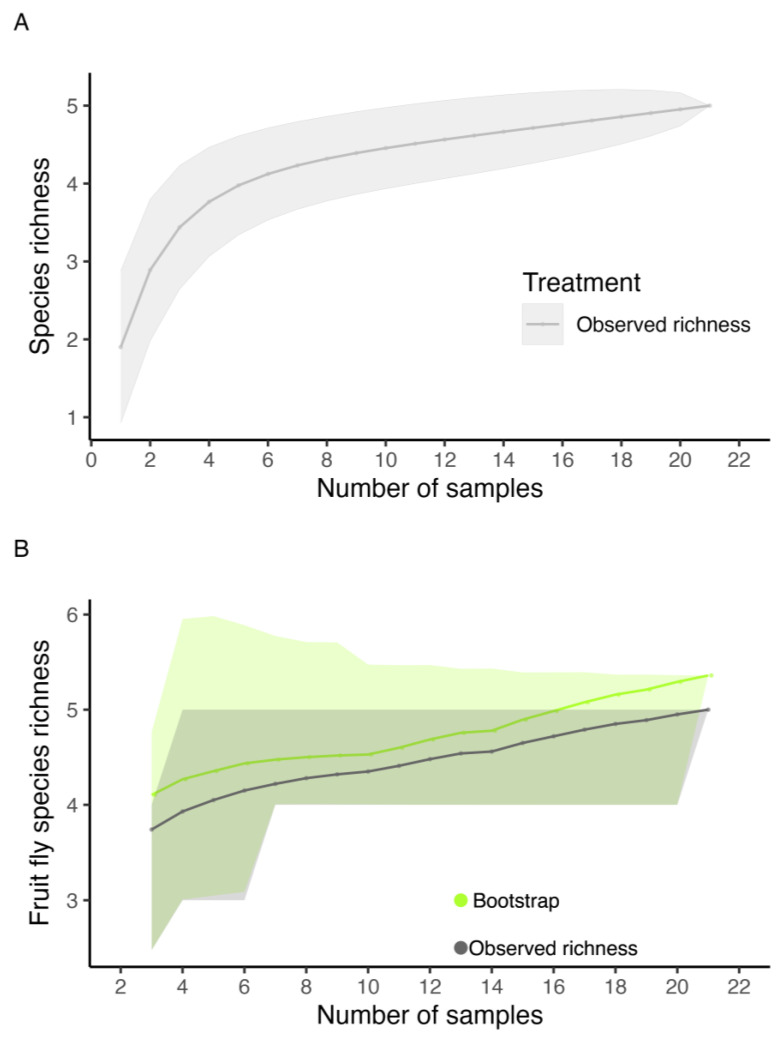
Coleman rarefaction curve and bootstrap richness estimator of fruit fly species (Diptera: Tephritidae) obtained from fruits collected in an experimental orchard of the Federal University of Lavras (UFLA), Minas Gerais, Brazil, during the period from February 2019 to June 2021. (**A**) Observed richness. (**B**) Observed richness and bootstrap richness estimator. The lines represent the observed and estimated richness. The shaded areas represent the standard error.

An asymptotic trend was observed in the species accumulation curve represented in the fruit samples, with a total observed richness of five fruit fly species (Figure 2). According to the bootstrap estimator (5.36), the species richness estimated for the orchard did not differ from the observed richness. According to the sampling effort, we obtained a species richness of 93.28% (Figure 2).

**Figure 3:**
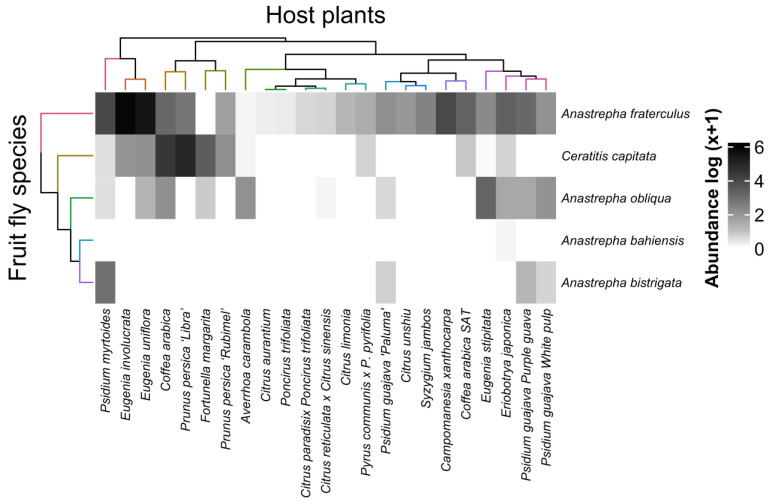
Heatmap of the interactions between fruit fly species (Diptera: Tephritidae) and host plants from the experimental orchard of the Federal University of Lavras (UFLA), Minas Gerais, Brazil. Darker tones indicate stronger interactions, and lighter tones indicate weaker interactions.

The interactions of fruit fly species with their fruiting hosts indicated that *A. fraterculus* interacted strongly with *Eugenia involucrata* Dc, followed by *Eugenia uniflora* L. and *Psidium myrtoides* O. Berg, and *C. capitata* showed the strongest interaction with *P. persica* cv. Libra and conventional coffee (Figure 3). Most interactions were strong, but weak interactions also occurred; for example, *A. bahiensis* interacted only with loquat (Figure 3).

#### 3.1.2. Parasitoids

We found that four species of the family Braconidae, *Doryctobracon areolatus* (Szépligeti), *Opius bellus* Gahan, *Utetes anastrephae* (Viereck) and *Asobara anastrephae* (Muesebeck), and three species of the family Figitidae, *Odontosema anastrephae* Borgmeier, *Aganaspis pelleranoi* (Brèthes) and *Lopheucoila anastrephae* (Rhower), were associated with fruit fly species obtained from host fruits. The parasitoid *D. areolatus* was obtained from samples of 14 fruit species, and this species was present in almost all of the fruits sampled. The species *O. bellus* and *U. anastrephae* were found in six species of fruit fly host fruits. *Asobara anastrephae*, in turn, was obtained from fruit flies infesting five fruit species. The species *A. pelleranoi* and *L. anastrephae* were found in samples of four fruit species, and *O. anastrephae* was associated with tephritids in three fruit species collected (Table 2).

**Figure 4:**
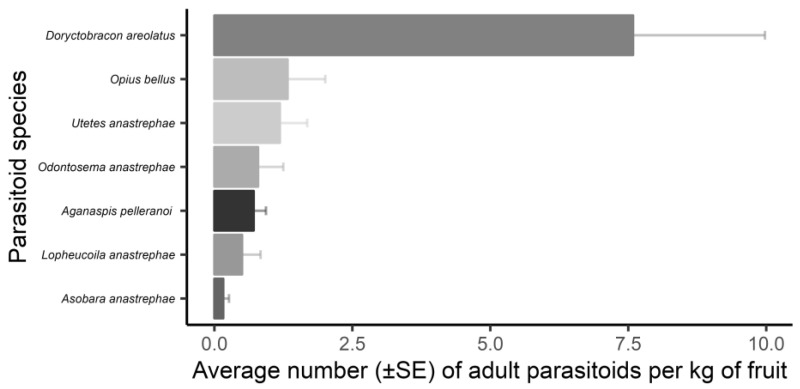
Mean numbers of fruit fly parasitoids per total fruit mass collected in an experimental orchard of the Federal University of Lavras (UFLA), Minas Gerais, Brazil, during the period February 2019 to June 2021. The bars represent the mean number of specimens captured, and the lines represent the standard error.

The composition of parasitoids obtained from fruit flies infesting fruits was similar, as there was no separation between the samples of fruits obtained from the trees and the soil by PERMANOVA analysis (*F*_1,28_ = 0.787; *p* < 0.602). Of the seven parasitoid species obtained from the samples, *D. areolatus* had the highest number of specimens per total fruit mass, followed by *O. bellus* and *U. anastrephae* (Figure 4).

**Figure 5:**
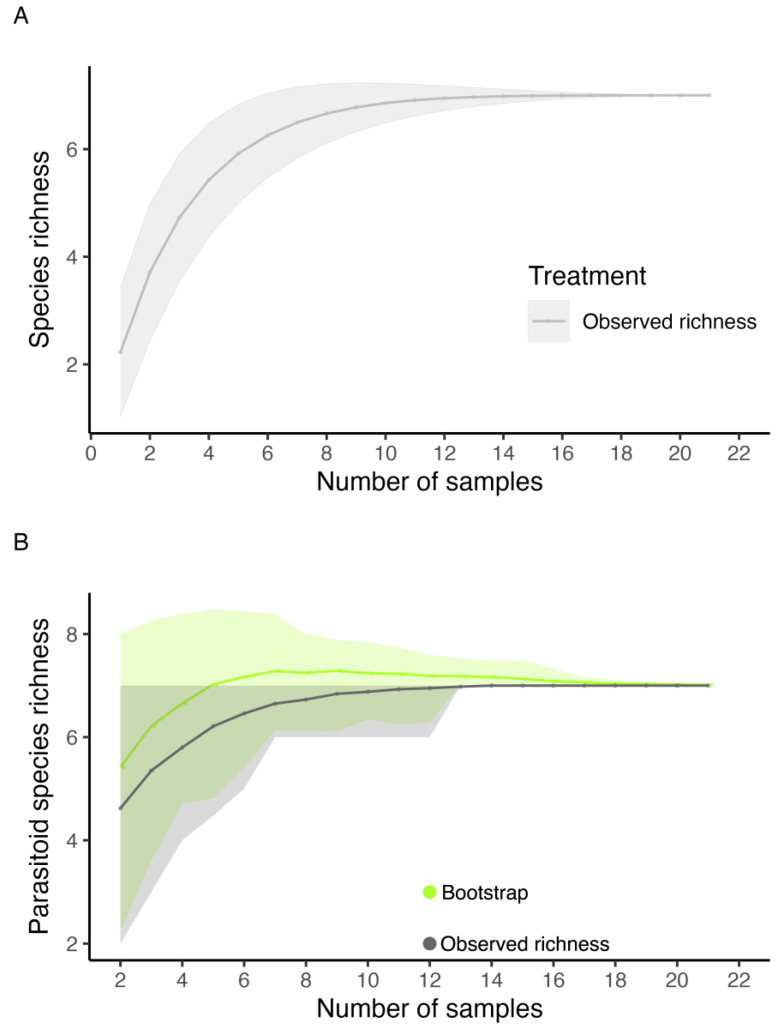
Coleman rarefaction curve and bootstrap richness estimator of parasitoid species obtained from fruit flies infesting fruits collected in an experimental orchard of the Federal University of Lavras (UFLA), Minas Gerais, Brazil, during the period of February 2019 to June 2021. (**A**) Observed richness. (**B**) Observed richness and bootstrap richness estimator. The lines represent the observed and estimated richness. The shaded areas represent the standard error.

There was stability in the accumulation curve of parasitoid species obtained from fruits infested by tephritids, with a total observed richness of seven species (Figure 5). The bootstrap estimator (7.02) indicated that we reached an expected richness of 98.59% of parasitoid species (Figure 5).

The percentage of parasitism varied depending on the host fruit species of fruit flies and among samples obtained from trees and soil (Table 4). The highest percentages of mean parasitism were observed in *E. uniflora* (49.89%), *P. myrtoides* (40.40%) and *C. xanthocarpa* (40.00%) (Table 3).

**Figure 6:**
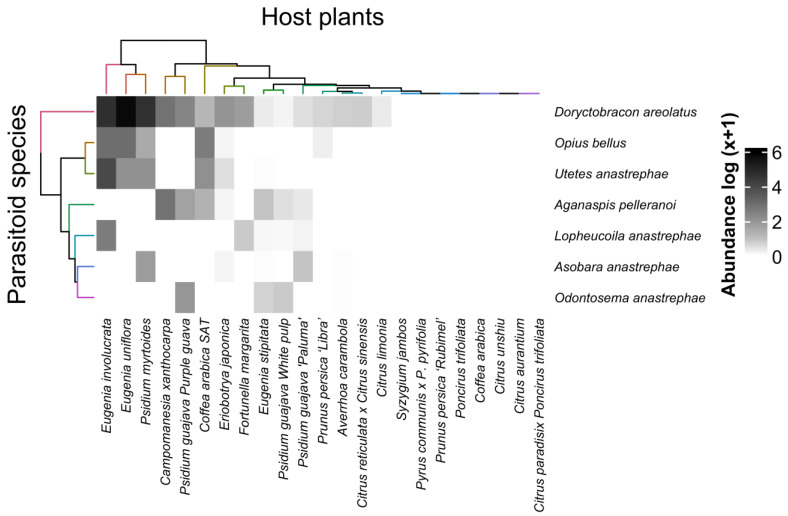
Heatmap of the interactions of parasitoid species with fruit infested by fruit flies from the experimental orchard of the Federal University of Lavras (UFLA), Minas Gerais, Brazil. Darker tones indicate stronger interactions, and lighter tones indicate weaker interactions.

The interactions of the parasitoid species obtained from the fruit samples infested by fruit flies showed that *D. areolatus* strongly interacted with *E. uniflora*, *P. myrtoides* and *E. involucrata* (Figure 6). Most interactions were weak; for example, *O. anastrephae* interacted only with *P. guajava* L. cv. Purple, *E. stipitata* and *P. guajava* cv. Pulp white.

### 3.2. Traps

Our sampling efforts resulted in the first records of *Anastrepha amita* Zucchi and *Anastrepha punctata* Hendel for the state of Minas Gerais. A total of 1768 female fruit flies of 10 species were collected. Among the species, *C. capitata*, *A. fraterculus* and *A. obliqua* were the most frequently collected. Males, as they were not used in identification, were excluded from the count (Table 4).

**Figure 7:**
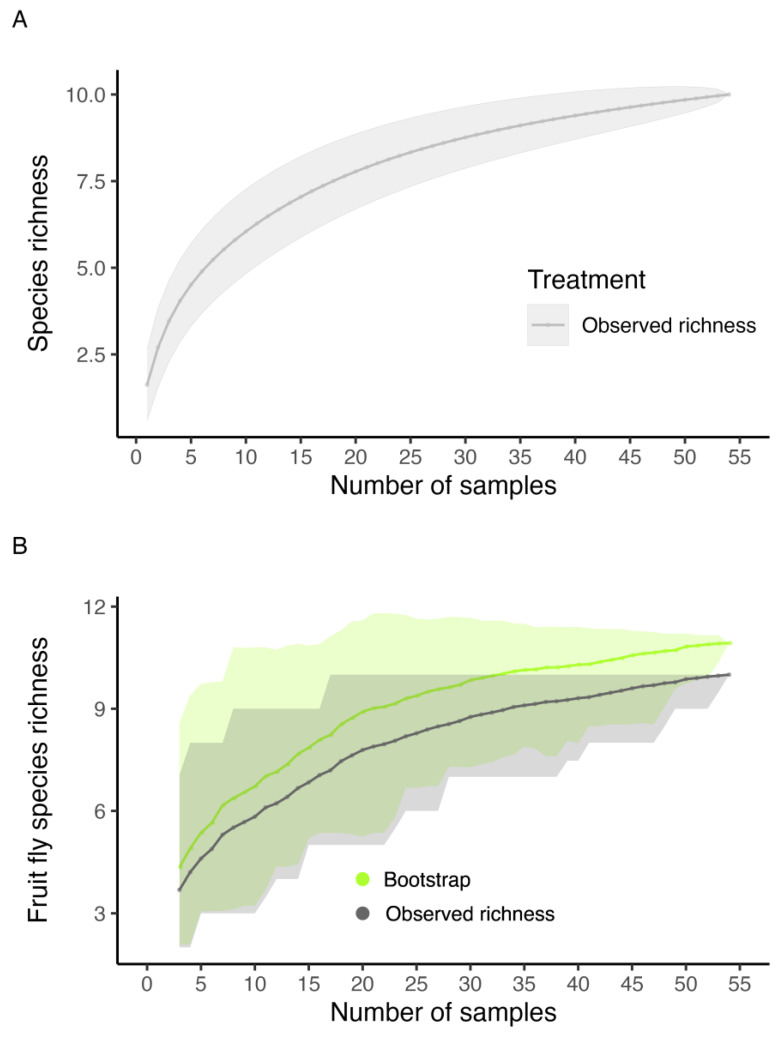
Coleman rarefaction curve and bootstrap richness estimator of in an experimental orchard of the Federal University of Lavras (UFLA), Minas Gerais, Brazil, from November 2019 to April 2021. (**A**) Observed richness. (**B**) Observed richness and bootstrap richness estimator. The lines represent the observed and estimated richness. The shaded areas represent the standard error.

An asymptotic trend was observed in the accumulation curve of species collected in the McPhail traps, with a total observed richness of 10 species (Figure 7). According to the bootstrap estimator, the species richness estimated for the orchard was not very different from the observed richness (10.90), indicating that we obtained an expected richness of 91.74% of fly species (Figure 7).

### 3.3. Population Fluctuation

**Figure 8:**
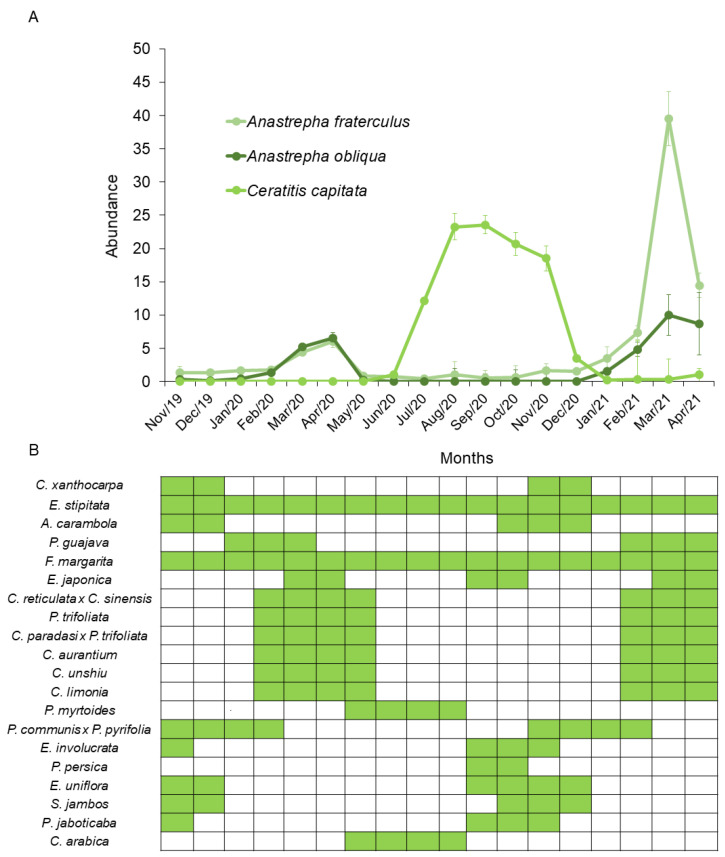
Population fluctuation of the most abundant fruit fly species collected using McPhail traps in an experimental orchard of the Federal University of Lavras (UFLA), Minas Gerais, Brazil, during the period from November 2019 to April 2021 (**A**) and temporal patterns of availability for host plant species throughout the year at the Federal University of Lavras (UFLA), Minas Gerais, Brazil (**B**).

The population fluctuation of fruit flies was calculated for the three most abundant species, *C. capitata*, *A. fraterculus* and *A. obliqua*. A peak in the populations of *A. fraterculus* and *A. obliqua* was observed from February 2020 to April 2020. Subsequently, there was a population peak for *C. capitata* in June 2020, which lasted until November, when few individuals of *Anastrepha* species were observed. In February 2021, another population peak of *A. fraterculus* and *A. obliqua* occurred, lasting until April (Figure 8).

## 4. Discussion

Currently, 36 species of the genus *Anastrepha* have been reported in Minas Gerais, Brazil [18,28]. The present study, however, contributes the first records of two more species, *A. amita* and *A. punctata*, bringing the total to 38 species. In addition, this article presents the results of the first survey on Tephritidae in a diversified orchard in southern Minas Gerais. Consequently, all the trophic relationships obtained here are new for this region and highlight the importance of expanding knowledge about the fruit fly complex in environments that are still underexplored and contain fruit diversity.

Trap and fruit sampling resulted in four shared species: *A. fraterculus*, *A. obliqua*, *A. bistrigata* and *C. capitata*. In general, the trap samples showed higher species richness than the fruit samples. This was mainly due to a greater sampling effort since the traps remained in the orchard continuously. Fruit collection, however, occurred according to the availability/fruiting period of the host species. Although the trap samples resulted in the highest species richness, observing the associations of hosts and their parasitoids was only possible through the collection of fruits [39]. Thus, both sampling methods were used in this study, which contributed to generating the information presented here.

Clearly, tephritid infestation is conditional on the intrinsic characteristics of the fruit species; thus, some of them seem to be strongly attractive, as is the case for species of the families Myrtaceae and Rosaceae (Table S1). *Anastrepha fraterculus*, for example, seems to prefer the species of the family Myrtaceae [24,25,39]. In addition, fruits of the family Myrtaceae, such as those of *P. guajava*, contributed to the presence of *A. bistrigata* in the orchard, which is similar to the findings of Araújo et al. [39]. However, some fruits may exhibit some degree of resistance to and/or non-preference for fruit flies. *Physalis peruviana* is, in this context, reported as a nonhost fruit for the Mediterranean fruit fly, *C. capitata*, either because the peel and resins/surface waxes prevent oviposition or, in the case of the larvae, because of the toxic compounds in the fruits [40]. Our findings add to this report, as no pupae were obtained from these fruits, and describe variations between different fruit trees (hosts or not, in addition to variations between infestation levels), supporting the need for surveys based on fruit sampling.

Here, we documented the first record of *P. myrtoides* as a host of *A. obliqua* in Brazil. In addition, new trophic associations were reported for the state of Minas Gerais. *Anastrepha bahiensis* was associated with *E. japonica* and *A. bistrigata* with *P. myrtoides* and *P. guajava*; to date, these are the only fruit hosts associated with these fly species in the state. On the other hand, *A. fraterculus and A. obliqua* infested the largest number of fruit species, and there was, in this study, an increase in the number of known hosts (Table 1). With these new occurrences, this number increased from 17 to 31 hosts associated with *A. fraterculus* and from 12 to 19 hosts associated with *A. obliqua*, recorded for Minas Gerais. The South American fruit fly, *A. fraterculus*, also interacted the most with the fruit species collected, with different intensities of interaction. However, the weakest interaction was observed for *A. bahiensis* in *E. japonica* (loquat), which is due to only one specimen being obtained.

*Ceratitis capitata* stands out for its adaptability [40], is among the most invasive species [19] and may prefer exotic fruit species. For Minas Gerais, there are 23 known hosts [20]. This study, however, revealed new associations between *C. capitata* and native Brazilian species, such as *E. stipitata*, *P. myrtoides* and *E. involucrata*. Overall, among native and exotic fruit trees, seven new species can be considered hosts of *C. capitata* in the state of Minas Gerais (Table 1). Interestingly, we did not obtain *C. capitata* adults from *P. guajava* fruits. In contrast, *P. persica* strongly influenced the presence of *C. capitata*, with a reduced number of specimens of *A. fraterculus* collected for this fruit tree. These species have probably developed strategies to explore distinct niches at different times of the year to avoid competition.

Seven species of parasitoids were obtained from fruits infested by fruit flies (Table 2). In addition, the highest rates of parasitism observed in this study in hosts such as *P. myrtoides* and *E. uniflora* (40.40–49.89%) suggest that the small size of the fruits may contribute to the effectiveness of parasitism. Although we did not find a correlation between parasitism and fruit size, it is a fact that in larger fruits, the tephritid host may become out of reach or escape by moving deeper inside the fruit, making it less susceptible to the parasitoid ovipositor [41]. Here, we classified *P. myrtoides* and *E. uniflora* as repositories of fruit fly parasitoids. In addition, this study confirmed that *D. areolatus* is the native parasitoid with the highest abundance, which is similar to what has been observed by other authors [15,22,24,42]. Among parasitoids frequently found attacking the same host, as in the case of *D. areolatus* and *U. anastrephae*, the size of the female ovipositor and the consequent access to the host may be an evolutionary adaptation that enables coexistence and provides a ’free space for competitors’. In this case, the longer ovipositor of *D. areolatus* may indicate a specialization that allows it to attack larvae in fruits larger than those accessible by *U. anastrephae*, which, in turn, is an intrinsically superior competitor to *D. areolatus* [43].

Regarding the species composition of the fruits sampled from trees and the ground, the samples similarly represented the fruit fly and parasitoid community. For parasitoids, for example, we recorded specimens that forage and attack their tephritid hosts while the fruits are still attached to the plant, which may explain the similarity between species compositions in tree- and ground-collected fruit. A difference could occur, however, if some of the collected species exhibited foraging activity/preference and parasitism in older tephritid hosts and, consequently, in fruits already present in the soil, as in the case of *Diachasmimorpha longicaudata* (Ashmead) (Hymenoptera: Braconidae) [44].

The population fluctuations of the most abundant species suggest a competitive hierarchy among *C. capitata*, *A. fraterculus* and *A. obliqua*. Therefore, the invasive species *C. capitata* could be displacing, at least in a certain period of the year, the species of *Anastrepha*. Previous reports of invasions involving these two genera suggest that *C. capitata* is competitively superior [45]. However, as previously mentioned, the highest abundance of *C. capitata* from June to November 2020 was mainly determined by the availability of *P. persica*, and the subsequent population peak of *A. fraterculus* may be, from this perspective, strongly associated with the availability of fruit from the Myrtaceae family. Hence, we can only state that population fluctuations between the two genera occurred due to different host availability throughout the collection period. Further studies will be necessary to investigate this possible displacement due to the occurrence of *C. capitata*.

Finally, the information obtained here paves the way for understanding some of the complex trophic relationships of fruit flies, host species and parasitoids in the state of Minas Gerais, especially in the southern region of the state. Future studies may explore other native host species in the search for new trophic associations that may contribute to and support integrated management strategies, such as the biological control of fruit flies in diverse environments.

## Figures and Tables

**Table 1:** Botanical family, host and fruit fly species, number of fruit flies emerged (females and males) and number of sampled fruits during the period from February 2019 to June 2021 (fruit orchard of UFLA, Lavras, MG, and domestic orchards in Itumirim, MG, and Ijaci, MG).

Hosts	Fruit Fly Species (Females)	Fruit Fly (Males)	Sampled Fruits (*n*)
Family/Species			
Myrtaceae			
*Eugenia stipitata*	*Anastrepha fraterculus* (Wied.) (177)	699	1373
	*Anastrepha obliqua* (Macquart) (496)		
	*Ceratitis capitata* (Wied.) (1)	0	
*Psidium myrtoides*	*Anastrepha bistrigata* Bezzi (58)	198	442
	*Anastrepha fraterculus* (Wied.) (178)		
	*Anastrepha obliqua* (Macquart) (2)		
	*Ceratitis capitata* (Wied.) (2)	4	
*Campomanesia xanthocarpa*	*Anastrepha fraterculus* (Wied.) (6)	1	7
*Psidium guajava* White pulp	*Anastrepha bistrigata* Bezzi (9)	152	291
	*Anastrepha fraterculus* (Wied.) (66)		
	*Anastrepha obliqua* (Macquart) (64)		
*Psidium guajava* White pulp *^a^*	*Anastrepha fraterculus* (Wied.) (25)	19	52
	*Anastrepha obliqua* (Macquart) (7)		
	*Anastrepha bistrigata* Bezzi (1)		
*Psidium guajava* ‘Paluma’	*Anastrepha bistrigata* Bezzi (5)	34	79
	*Anastrepha fraterculus* (Wied.) (36)		
	*Anastrepha obliqua* (Macquart) (4)		
*Psidium guajava* Purple guava	*Anastrepha bistrigata* Bezzi (17)	172	377
	*Anastrepha fraterculus* (Wied.) (162)		
	*Anastrepha obliqua* (Macquart) (26)		
*Plinia jaboticaba*	*Anastrepha fraterculus* (Wied.) (1)	1	2
*Eugenia involucrata*	*Anastrepha fraterculus* (Wied.) (110)	107	222
	*Ceratitis capitata* (Wied.) (2)	3	
*Syzygium jambos*	*Anastrepha fraterculus* (Wied.) (5)	3	16
	*Ceratitis capitata* (Wied.) (0)	8	
*Eugenia uniflora*	*Anastrepha fraterculus* (Wied.) (99)	128	232
	*Anastrepha obliqua* (Macquart) (1)		
	*Ceratitis capitata* (Wied.) (3)	1	
*Eugenia uniflora ^a^*	*Anastrepha fraterculus* (Wied.) (31)	35	66
*Eugenia pyriformis ^a^*	*Anastrepha obliqua* (Macquart) (53)	31	84
Rubiaceae			
*Coffea arabica*	*Anastrepha fraterculus* (Wied.) (3)	0	31
	*Anastrepha obliqua* (Macquart) (1)		
	*Ceratitis capitata* (Wied.) (12)	15	
*Coffea arabica* SAT *^c^*	*Anastrepha fraterculus* (Wied.) (77)	83	167
	*Ceratitis capitata* (Wied.) (4)	3	
Oxalidaceae			
*Averrhoa carambola*	*Anastrepha fraterculus* (Wied.) (6)	138	422
	*Anastrepha obliqua* (Macquart) (263)		
	*Ceratitis capitata* (Wied.) (7)	8	
Rutaceae			
*Citrus paradisi* × *Poncirus trifoliata*	*Anastrepha fraterculus* (Wied.) (2)	1	3
*Citrus reticulata* × *Citrus sinensis*	*Anastrepha fraterculus* (Wied.) (6)	4	11
	*Anastrepha obliqua* (Macquart) (1)		
*Citrus aurantium*	*Anastrepha fraterculus* (Wied.) (1)	2	3
*Citrus sinensis ^b^*	*Anastrepha fraterculus* (Wied.) (4)	2	6
*Fortunella margarita*	*Anastrepha obliqua* (Macquart) (2)	10	78
	*Ceratitis capitata* (Wied.) (49)	17	
*Citrus limonia*	*Anastrepha fraterculus* (Wied.) (6)	7	13
*Poncirus trifoliata*	*Anastrepha fraterculus* (Wied.) (3)	1	4
*Citrus unshiu*	*Anastrepha fraterculus* (Wied.) (9)	7	16
Rosaceae			
*Eriobotrya japonica*	*Anastrepha fraterculus* (Wied.) (173)	217	486
	*Anastrepha obliqua* (Macquart) (23)		
	*Anastrepha bahiensis* Lima (1)		
	*Ceratitis capitata* (Wied.) (6)	66	
*Pyrus communis* × *P. pyrifolia*	*Anastrepha fraterculus* (Wied.) (9)	15	32
	*Ceratitis capitata* (Wied.) (3)	5	
*Prunus persica* ‘Libra’	*Anastrepha fraterculus* (Wied.) (52)	48	1124
	*Ceratitis capitata* (Wied.) (496)	528	
*Prunus persica* ‘Rubimel’	*Anastrepha fraterculus* (Wied.) (5)	4	25
	*Ceratitis capitata* (Wied.) (8)	8	

*^a^* Collections performed in domestic orchards in Ijaci, MG; *^b^* Collection performed in a domestic orchard in Itumirim, MG; *^c^ Coffea arabica* SAT: coffee without pesticide applications.

**Table 2:** Species of parasitoids and their host fruit flies obtained from fruits collected during the period from February 2019 to June 2021 (fruit orchard of UFLA, Lavras, MG, and domestic orchards in Itumirim, MG, and Ijaci, MG).

Species of Parasitoids	Number	Associated Plants	Host Fruit Flies
*Aganaspis pelleranoi* (Brèthes)	31	*Eugenia stipitata*	*A. fraterculus*, *A. obliqua*, *C. capitata*
	2	*Campomanesia xanthocarpa*	*A. fraterculus*
	6	*Psidium guajava* White pulp	*A. bistrigata*, *A. fraterculus*, *A. obliqua*
	2	*P. guajava* ‘Paluma’	*A. bistrigata*, *A. fraterculus*, *A. obliqua*
	32	*P. guajava* Purple guava	*A. bistrigata*, *A. fraterculus*, *A. obliqua*
	8	*Coffea arabica* SAT *^b^*	*A. fraterculus*, *C. capitata*
*Doryctobracon areolatus* (Szépligeti)	7	*E. stipitata*	*A. fraterculus*, *A. obliqua*, *C. capitata*
	344	*Psidium myrtoides*	*A. bistrigata*, *A. fraterculus*, *A. obliqua*, *C. capitata*
	2	*C. xanthocarpa*	*A. fraterculus*
	2	*P. guajava* White pulp	*A. bistrigata*, *A. fraterculus*, *A. obliqua*
	3	*P. guajava* ‘Paluma’	*A. bistrigata*, *A. fraterculus*, *A. obliqua*
	73	*P. guajava* Purple guava	*A. bistrigata*, *A. fraterculus*, *A. obliqua*
	35	*Eugenia involucrata*	*A. fraterculus*, *C. capitata*
	133	*Eugenia uniflora*	*A. fraterculus*, *A. obliqua*, *C. capitata*
	48	*Eugenia uniflora ^a^*	*A. fraterculus*
	1	*Eugenia pyriformis ^a^*	*A. obliqua*
	7	*C. arabica* SAT *^b^*	*A. fraterculus*, *C. capitata*
	40	*Averrhoa carambola*	*A. fraterculus*, *A. obliqua*, *C. capitata*
	7	*Citrus reticulate* × *Citrus sinensis*	*A. fraterculus*, *A. obliqua*
	8	*Fortunella margarita*	*A. obliqua*, *C. capitata*
	1	*Citrus limonia*	*A. fraterculus*
	43	*Eriobotrya japonica*	*A. fraterculus*, *A. obliqua*, *A. bahiensis*, *C. capitata*
	3	*Prunus persica* ‘Libra’	*A. fraterculus*, *C. capitata*
*Lopheucoila anastrephae* (Rhower)	3	*E. stipitata*	*A. fraterculus*, *A. obliqua*, *C. capitata*
	1	*P. guajava* White pulp	*A. bistrigata*, *A. fraterculus*, *A. obliqua*
	1	*P. guajava* ‘Paluma’	*A. bistrigata*, *A. fraterculus*, *A. obliqua*
	4	*E. involucrata*	*A. fraterculus*, *C. capitata*
	2	*F. margarita*	*A. obliqua*, *C. capitata*
*Odontosema anastrephae* Borgmeier	21	*E. stipitata*	*A. fraterculus*, *A. obliqua*, *C. capitata*
	12	*P. guajava* White pulp	*A. bistrigata*, *A. fraterculus*, *A. obliqua*
	45	*P. guajava* Purple guava	*A. bistrigata*, *A. fraterculus*, *A. obliqua*
	1	*A. carambola*	*A. fraterculus*, *A. obliqua*, *C. capitata*
*Opius bellus* Grahan	11	*P. myrtoides*	*A. bistrigata*, *A. fraterculus*, *A. obliqua*, *C. capitata*
	6	*E. involucrata*	*A. fraterculus*, *C. capitata*
	8	*E. uniflora*	*A. fraterculus*, *A. obliqua*, *C. capitata*
	6	*E. uniflora ^a^*	*A. fraterculus*
	38	*C. arabica* SAT *^b^*	*A. fraterculus*, *C. capitata*
	1	*E. japonica*	*A. fraterculus*, *A. obliqua*, *A. bahiensis*, *C. capitata*
	1	*P. persica* ‘Libra’	*A. fraterculus*, *C. capitata*
*Utetes anastrephae* (Viereck)	1	*E. stipitata*	*A. fraterculus*, *A. obliqua*, *C. capitata*
	23	*P. myrtoides*	*A. bistrigata*, *A. fraterculus*, *A. obliqua*, *C. capitata*
	16	*E. involucrata*	*A. fraterculus*, *C. capitata*
	3	*E. uniflora*	*A. fraterculus*, *A. obliqua*, *C. capitata*
	8	*E. uniflora ^a^*	*A. fraterculus*
	22	*C. arabica* SAT *^b^*	*A. fraterculus*, *C. capitata*
	4	*E. japonica*	*A. fraterculus*, *A. obliqua*, *A. bahiensis*, *C. capitata*
*Asobara anastrephae* (Muesebeck)	1	*E. stipitata*	*A. fraterculus*, *A. obliqua*, *C. capitata*
	17	*P. myrtoides*	*A. bistrigata*, *A. fraterculus*, *A. obliqua*, *C. capitata*
	7	*P. guajava* ‘Paluma’	*A. bistrigata*, *A. fraterculus*, *A. obliqua*
	2	*A. carambola*	*A. fraterculus*, *A. obliqua*, *C. capitata*
	1	*E. japonica*	*A. fraterculus*, *A. obliqua*, *A. bahiensis*, *C. capitata*

*^a^* Collections performed in domestic orchards in Ijaci, MG; *^b^ Coffea arabica* SAT: coffee without pesticide applications.

**Table 3:** Host plants and rate of parasitism of fruits infested by fruit flies (Diptera: Tephritidae) sampled from the trees and/or the ground, during the period February 2019 to June 2021 (Fruit Culture Orchard of UFLA, MG, and domestic orchards in Itumirim, MG, and Ijaci, MG).

Hosts	Parasitism (%)		Mean Parasitism (%) *^1^*
Family/Species	Tree	Ground	
Myrtaceae			
*Eugenia stipitata*	1.29	3.99	2.84
*Psidium myrtoides*	32.73	61.86	40.4
*Campomanesia xanthocarpa*	0	80	40
*Psidium guajava* White pulp	0.71	13.65	7.18
*P. guajava* Whitepulp *^a^*	0	0	0
*P. guajava* Purple guava	21.02	37.5	28.51
*Plinia jaboticaba*	0	0	0
*Eugenia involucrata*	15.37	40.9	27
*Syzygium jambos*	0	0	0
*Eugenia uniflora*	36.66	38.81	37.73
*E. uniflora ^a^*	40.54	59.25	49.89
*Eugenia pyriformis*	0.9	0	0.45
Rutaceae			
*Citrus paradisi* × *Poncirus trifoliata*	0	0	0
*Citrus sinensis*	0	0	0
*Fortunella margarita*	0.55	54.68	30.62
*Citrus limonia*	0	7.14	3.57
*Poncirus trifoliata*	0	0	0
*Citrus unshiu*	0	0	0
Rubiaceae			
*Coffea arabica*	0	-	0
*C. arabica* SAT *^b^*	31.4	-	31.4
Oxalidaceae			
*Averrhoa carambola*	6.96	7.09	7.02
Rosaceae			
*Eriobotrya japonica*	13.65	9.55	11.6
*Pyrus communis* × *P. pyrifolia*	0	0	0
*Prunus persica* ‘Libra’	0.3	0.28	0.19
*P. persica* ‘Rubimel’	0	0	0

*^1^* The mean parasitism (%) was calculated considering the total parasitism, obtained from trees and the ground; *^a^* Collections performed in domestic orchards in Ijaci, MG; *^b^ Coffea arabica* SAT: coffee without pesticide applications.

**Table 4:** Fruit flies collected with McPhail traps during the period from November 2019 to April 2021 (UFLA fruit orchard, Lavras, MG).

Species	Females (*n*)	Collections (*n*)	Relative Frequency
*Ceratitis capitata*	880	22	0.498
*Anastrepha fraterculus*	600	45	0.339
*Anastrepha obliqua*	255	24	0.144
*Anastrepha distincta*	11	8	0.006
*Anastrepha grandis*	7	7	0.004
*Anastrepha chiclayae*	6	4	0.003
*Anastrepha bistrigata*	4	4	0.002
*Anastrepha pseudoparallela*	3	2	0.002
*Anastrepha amita*	1	1	0.001
*Anastrepha punctata*	1	1	0.001
Total	1768		
Richness S’	10		

## Data Availability

Data are available upon request.

## Supplementary Materials

The following supporting information can be downloaded at: https://www.mdpi.com/article/10.3390/insects16010017/s1, Figure S1: Climatic data obtained from the experimental orchard of the Federal University of Lavras (UFLA), Minas Gerais, Brazil, during the period February 2019 to April 2021; Figure S2: Geographical location of McPhail traps in the experimental orchard of the Federal University of Lavras (UFLA), Minas Gerais, Brazil, during the period November 2019 to April 2021; Table S1: Botanical family, scientific and vernacular names, number and weight of fruits sampled, classified as infested or not infested by fruit flies (Diptera: Tephritidae), during the period from February 2019 to June 2021. (Orchard de UFLA, Lavras, MG, and domestic orchards in Itumirim, MG, and Ijaci, MG).
