# Supplementary material for: Fruit Flies (Diptera: Tephritidae) in Minas Gerais, Brazil: Trophic Interactions and New Reports
Source: Insects. 2024 Dec 28;16(1):17. doi: 10.3390/insects16010017 (PMC11766348; doi:10.3390/insects16010017)
Supplement: Supplementary file 1 [file insects-16-00017-s001.zip › insects-3359371-supplementary.pdf]

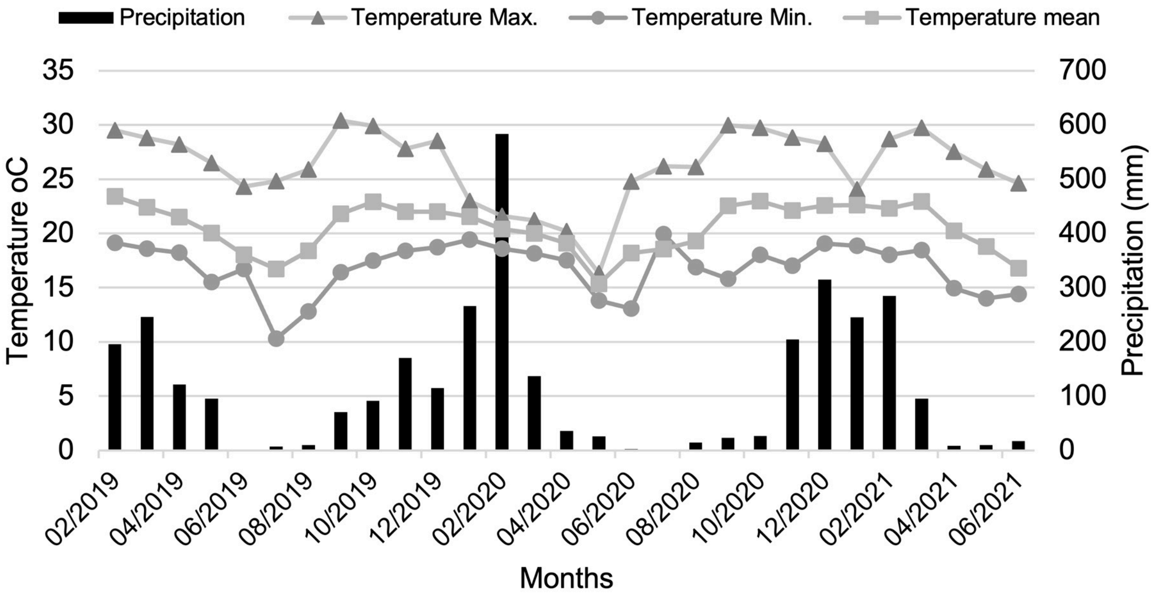

Figure S1. Climatic data obtained from the experimental orchard of the Federal University of Lavras - UFLA, Minas Gerais, Brazil, during the period February 2019 to April 2021.

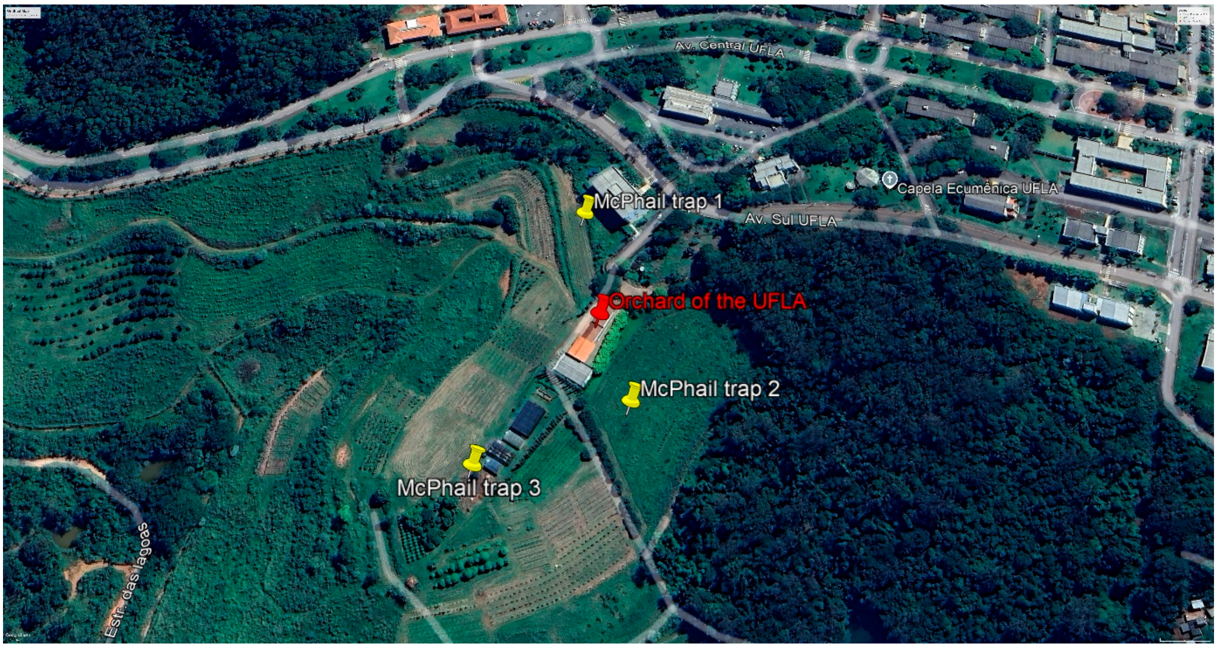

Figure S2. Geographical location of McPhail traps in the experimental orchard of the Federal University of Lavras - UFLA, Minas Gerais, Brazil, during the period November 2019 to April 2021.

Table S1. Botanical family, scientific and vernacular names, number and weight of fruits sampled, classified as infested or not infested by fruit flies (Diptera: Tephritidae), during the period from February 2019 to June 2021. (Orchard de UFLA, Lavras, MG and domestic orchards in Itumirim, MG and Ijaci, MG).

Plants		Number of fruits (n)	Weight (kg)	Infested fruit Yes/No	Infestation index		Infestation index
Family/Species	Common name				Tree	Soil	
**Myrtaceae**							
Eugenia stipitata MacVaugh	Araçá-boi	298 (16)	20.36	Yes	11.55	4.76	7.67
Psidium myrtoides O. Berg	Purple araçá	964 (13)	3.24	Yes	1.69	0.95	1.49
Eugenia involucrata DC.	Rio Grande cherry	203 (2)	0.31	Yes	1.69	1.76	1.72
Campomanesia xanthocarpa O. Berg	Gabiroba	40 (1)	0.12	Yes	0.35	0.35	0.35
Psidium guajava L.	White pulp guava	267 (7)	10.67	Yes	3.09	1.62	2.35
	White pulp guava[a]	30 (2)	1.58	Yes	3.66	1.61	2.29
	'Paluma'	40 (4)	4.73	Yes	6.72	2.67	5.71
	Purple guava	154 (6)	8.1	Yes	9.01	2.98	6.27
Plinia jaboticaba (Vell.) Kausel	Jaboticaba[a]	127 (1)	0.27	Yes	0.05	0.05	0.05
Syzygium jambos (L.) Alston	Rose apple	11 (1)	0.47	Yes	3.33	0	1.67
Eugenia uniflora L.	Pitanga	110 (1)	0.41	Yes	3.96	3.38	3.67
	Pitanga[a]	100 (1)	0.23	Yes	1.54	1.09	1.31
Eugenia pyriformis Cambess	Uvaia[a]	45 (3)	0.82	Yes	3.15	1.8	2.47
**Rubiaceae**							

Continue...

Table S1. Continuation.

Plants		Number of fruits (n)	Weight (kg)	Infested fruit Yes/No	Infestation index		Infestation index
Family/Species	Common name				Tree	Soil	
Coffea arabica L.	Conventional coffee	105 (1)	0.13	Yes	0.3	-	0.3
	Coffee SAT[c]	2,757 (3)	3.01	Yes	0.16	-	0.16
**Oxalidaceae**							
Averrhoa carambola L.	Star fruit	654 (16)	36.82	Yes	1.43	1.12	1.28
**Rutaceae**							
Citrus paradisi (L.) Osb. x *Poncirus trifoliata* (L.) Raf.	Citrange	15 (1)	2.45	Yes	0.2	-	0.2
Citrus paradisi Macfad. x *Poncirus trifoliata* L.) Raf.	Citrumelo	12 (1)	1.56	Yes	0.08	-	0.08
Citrus reticulata Blanco. x *Citrus sinensis* (L.) Osbeck	Dekopon tangerine	70 (6)	7.15	Yes	0.6	1.43	0.72
Citrus aurantium L.	Sour orange	19 (1)	3.22	Yes	-	0.16	0.16
Citrus sinensis (L.)	Serra d'água orange[b]	5 (1)	0.84	Yes	1.8	-	1.8
Fortunella margarita (Lour.) Swingle	Kinkan orange	162 (5)	1.96	Yes	0.85	0.5	0.65
Citrus limonia Osbeck	Rangpur lime	66 (3)	3.6	Yes	0.57	0.43	0.5
Poncirus trifoliata (L.)	'Flying Dragon'	240 (2)	10.36	No	-	-	-
	Poncirus	47 (2)	8.6	Yes	0.06	0.23	0.17
Citrus unshiu (Mak.) Marcov.	Tangerine satsuma	20 (2)	1.48	Yes	2.22	3	2.61

Continue...

Table S1. Continuation.

Plants		Number of fruits (n)	Weight (kg)	Infested fruit Yes/No	Infestation index		Infestation index
Family/Species	Common name				Tree	Soil	
**Rosaceae**							
Eriobotrya japonica (Thunb.) Lindl.	Loquat	586 (11)	6.66	Yes	0.98	1.01	0.99
Pyrus communis x *P. pyrifolia*	Pear	50 (2)	2.95	Yes	0.95	0.67	0.86
Prunus persica L.	'Libra' peach	157 (3)	3.32	Yes	9.32	4.14	6.73
	'Rubimel' peach	23 (1)	1.06	Yes	1.13	-	1.13
Rubus niveus Thunb.	Black raspberry	211 (1)	0.21	No	-	-	-
	Red raspberry	180 (3)	0.49	No	-	-	-
**Solanaceae**							
Solanum betaceum Cav.	Tamarillo	162 (14)	6.19	No	-	-	-
Physalis peruviana L.	Physalis	128 (4)	0.35	No	-	-	-
**Cactaceae**							
Selenicereus undatus (Haw.)	White pitaya	24 (4)	4.91	No	-	-	-
Selenicereus polyrhizus	Red pitaya	5 (1)	1.3	No	-	-	-

Continue...

Table S1. Continuation.

Plants		Number of fruits (n)	Weight (kg)	Infested fruit Yes/No	Infestation index		Infestation index
Family/Species	Common name				Tree	Soil	
**Moraceae**							
Ficus carica L.	Fig	38 (2)	2.94	No	-	-	-
**Passifloraceae**							
Passiflora edulis Sims.	Passion fruit	21 (1)	3.84	No	-	-	-

(n) Number of samples collected from each species
[a]Collections performed in domestic orchards of Ijaci, MG
[b]Collection performed in the domestic orchard of Itumirim, MG
[c]Coffee SAT: coffee without pesticide applications
